# Super paramagnetic clustering of protein sequences

**DOI:** 10.1186/1471-2105-6-82

**Published:** 2005-04-01

**Authors:** Igor V Tetko, Axel Facius, Andreas Ruepp, Hans-Werner Mewes

**Affiliations:** 1 GSF National Research Center for Environment and Health, Institute for Bioinformatics, Ingolstädter Landstraße 1, D-85764 Neuherberg, Germany; 2 IBPC, Biomedical Department, Ukrainian Academy of Sciences, Murmanskaya, 1, UA-02094, Kyiv, Ukraine; 3 Department of Genome-Oriented Bioinformatics, Wissenschaftszentrum Weihenstephan, Technische Universität München, 85350 Freising, Germany

## Abstract

**Background:**

Detection of sequence homologues represents a challenging task that is important for the discovery of protein families and the reliable application of automatic annotation methods. The presence of domains in protein families of diverse function, inhomogeneity and different sizes of protein families create considerable difficulties for the application of published clustering methods.

**Results:**

Our work analyses the Super Paramagnetic Clustering (SPC) and its extension, global SPC (gSPC) algorithm. These algorithms cluster input data based on a method that is analogous to the treatment of an inhomogeneous ferromagnet in physics. For the SwissProt and SCOP databases we show that the gSPC improves the specificity and sensitivity of clustering over the original SPC and Markov Cluster algorithm (TRIBE-MCL) up to 30%. The three algorithms provided similar results for the MIPS FunCat 1.3 annotation of four bacterial genomes, *Bacillus subtilis*, *Helicobacter pylori*, *Listeria innocua *and *Listeria monocytogenes*. However, the gSPC covered about 12% more sequences compared to the other methods. The SPC algorithm was programmed in house using C++ and it is available at . The FunCat annotation is available at .

**Conclusion:**

The gSPC calculated to a higher accuracy or covered a larger number of sequences than the TRIBE-MCL algorithm. Thus it is a useful approach for automatic detection of protein families and unsupervised annotation of full genomes.

## Background

Numerous genome-sequencing projects have caused a rapid growth of the protein databases. In contrast to the pre-genomic era, when the selection of sequences was highly biased towards known and characterized genes, the systematic exploration of genomes now allows to assign more and precise functional properties in the majority of cases. However, manual annotation of sequences is laborious and expensive. Thus, there is a strong interest in developing reliable methods for the automatic functional classification of genome sequences employing evolutionary sequences as reflected in using sequence homology to predict functional properties. The identification of protein families, defined as set of proteins with significant sequence similarity encoding for at least related but often identical function between members, is a very important subtask to achieve this fundamental goal. Indeed, the fact that proteins with high sequence similarity share a common evolutionary history is accepted as the basis for functional assignment [1].

Among the different methods proposed to organize the sequence space into protein families, several approaches based on clustering using sequence similarity scores were successfully established (see e.g., [2-4]). However, the multi-domain composition of proteins, as well as the presence of promiscuous domains can influence the accuracy of such methods. Recently, an efficient algorithm for large-scale detection of protein families based on the Markov cluster algorithm, TRIBE-MCL, was proposed [5]. This algorithm simulates random walks within a graph by iterative alternation of two operators called expansion (explores intra-cluster structure) and inflation (eliminates flow between different clusters). In comparison to other clustering algorithms, the TRIBE-MCL produces clusters that resist contamination by promiscuous domains that could provide significant problems for other clustering algorithms as is discussed elsewhere [6]. TRIBE-MCL was tested using large databases of manually annotated protein sequences such as SwissProt [7] and SCOP [8] and has already been widely used in bioinformatics (about 50 publications referred to this algorithm since its publication in 2002[5] according to [9]). Thus this method is one of the recognized bioinformatic tools and its results can be used as an established standard for comparison of new algorithms. Moreover, we have already used the TRIBE-MCL algorithm for the analysis of the SIMAP database [10].

Several clustering methods have appeared in recent years. One of these, the Super Paramagnetic Clustering (SPC) has received considerable attention in microarray data analysis [11,12]. This algorithm provides clustering of input data [13] based on analogy to the physics of an inhomogeneous ferromagnet. The method detects natural (physical) clusters present in the data and is able to efficiently cluster difficult test examples, such as concentric circles. The SPC algorithm was also successfully used in a supervised setting to analyze protein sequences and classify SCOP and CATH proteins according to their FSSP scores [14]. Following our first successful application of SPC to a database of RING-finger domains [15] and our approach to project expression data to known functional modules [16], the present study further investigates the power of SPC to cluster protein sequences of two large databases, SwissProt and SCOP. We compare its performance with the TRIBE-MCL algorithm. Since both these databases do not contain complete genome sequences required for an unbiased comparison of the methods, we additionally analyzed protein sequences from four bacterial genomes, namely *Bacillus subtilis*, *Helicobacter pylori*, *Listeria innocua *and *Listeria monocytogenes *manually annotated at MIPS according to FunCat [17-19]. We also introduce an extension of this algorithm, global SPC or gSPC, which performs step-wise clustering on different levels of connectivity between points and provides significantly improved performance to the annotation of whole genomes compared to both the original SPC algorithm and TRIBE-MCL.

## Results

### Clustering of a simulated data

**Figure 1:**
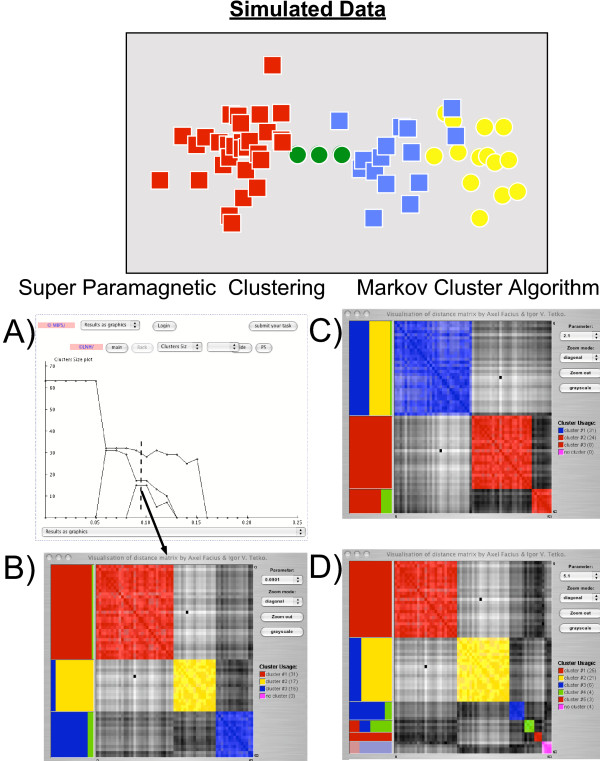
Clustering of Artificial Data Set using SPC and TRIBE-MCL algorithm. The red, blue and yellow colors correspond to three distributions used to generate the data. The noise between the data points from distributions 1 and 2 is indicated as green circles. A) Cluster size plot of SPC algorithm. The vertical line indicates temperature when calculation of the distance matrix B) was performed. B) The distance matrix calculated for the SPC clusters at temperature *T *= 0.087. More intense colors correspond to smaller distances between points. The diagonal and off-diagonal elements correspond to inter- and intra- cluster distances, respectively. Each horizontal block on the left side of Figure corresponds to one cluster and the colors are used to indicate composition of samples from different distributions. C) and D) are the distance matrices calculated for the TRIBE-MCL clusters obtained using inflation parameter 2.1 and 5.1, respectively.

We tested the ability of the SPC method to determine the physical number of clusters in the data using synthetic data. The model problem of Figure 1 consisted of *n *= 60 points in *D *= 2 dimensions. The data points were generated using three normal distributions *N*(**x**_**i**_, σ = 1.5), with centers **x**_**0**_(1; 2) (*n *= 60 samples), **x**_**1**_(10; 2) (*n *= 30) and **x**_**2**_(14; 2) (*n *= 30). The data points generated by the second and third normal distributions are overlapping. In addition three points, **x **= (4.5 + 1.5*j;2), j = {0,1,2} were added to simulate an artificial link between the data points from the *1*^*st *^and *2*^*nd *^distributions.

Figure 1 demonstrates that SPC (*K *= 10) was able to correctly determine the presence of three clusters in the data. Two splits of clusters at temperature *T *= 0.054 and *T *= 0.084 are observed. The first split corresponds to a separation of clusters formed by the *1*^*st *^and two other distributions. The second split corresponds to a separation of clusters formed by *2*^*nd *^and *3*^*rd *^distributions. Following these two breaks, the cluster melts on singletons. Thus, the hierarchical structure of data was uncovered and physical clusters present in these data were found. The noise between the data points from distributions 1 and 2 (green circles) did not affect the clustering results.

In contrast to SPC, the TRIBE-MCL algorithm has some difficulties in correctly determine the structure of the data. For example, for inflation parameter 2.1, the algorithm subclusters points generated by normal distributions 1 into 2 different subclusters. For the inflation value of 5, one can already observe 5 different clusters. One of the largest clusters contains 21 points, including 6 and 15 points generated by distribution 2 and 3, respectively. This cluster remains stable even for inflation parameter 20. Thus TRIBE-MCL could not detect the physical structure of this data set. Of course, one should not draw a general conclusion about the relative performance of both algorithms following only a single simulated example.

### Comparison of algorithms using SwissProt and SCOP databases

The performance of the algorithms was investigated using SwissProt [7] and SCOP [3,8] databases. The accuracy of SPC clustering for the SwissProt database was assessed by analysis of InterPro domains [20] and Swiss keywords of members in calculated clusters. Sequences without any annotation were used for data clustering but were not considered to estimate the performance of the methods. Ideally, all members of each detected cluster should have exactly the same annotation in terms of InterPro domains and Swiss keywords.

Analogous to the previous analysis [5] only clusters that contained at least 4 or more annotated sequences were considered. The domain (or keyword) combination detected for ≥50% annotated sequences in the cluster was used as the (consensus) annotation of the cluster. Since some proteins had more than one domain (or keyword), we measured the performance of the method by counting the number of correctly assigned domains rather than the number of correctly classified proteins. This procedure avoids ambiguities in cases where, for instance, the annotation of three out of five domains was predicted correctly. The number of true positive (*TP*) domains/keywords was determined as the count of domains/keywords that coincided with the cluster annotation. The number of false negatives (*FN*), i.e. domains/keywords observed for a particular protein but absent in the cluster annotation, and false positive (*FP*), i.e. domains/keywords presented in the cluster annotation but missed for some particular proteins, were calculated. These numbers were used to compute the sensitivity = *TP*/(*TP *+ *FN*) and specificity = *TP*/(*TP *+ *FP*) of the clustering algorithms analyzed. The sensitivity is equivalent to the probability of correctly predicting some classifier while specificity is defined as the probability that the provided prediction is correct [21].

Not all proteins initially used in the evaluation will get a chance to be annotated by clustering. Some proteins will not be clustered at all, because they do not have significant hits to other proteins. These proteins can be either treated as false negatives (indeed, their categories were not predicted) or simply excluded from the analysis (since they cannot be clustered and the clustering algorithm explicitly "refused" to annotate them). The sensitivity that is calculated taking clustered proteins appears to be more relevant. Indeed, if a protein was clustered, the sensitivity determined in our study will indicate how many of the existing categories of the protein analyzed are expected to be correctly predicted. This definition deals with *a posteriori *sensitivity, i.e. it should be used only after clustering of the protein families. The sensitivity determined by considering all non-clustered proteins as false positive, corresponds to *a priori *sensitivity. Indeed, this number indicates how many categories of the given protein will be correctly predicted when there is no knowledge if a protein will be clustered or not. Since each of these two definitions of sensitivity has its own advantage (e.g. the later allows for a more straightforward comparison of methods) we calculated them both. Notice, that this definition of specificity does not include *FN *and thus it is not affected by which definition is used.

### SCOP database analysis

Sequences from the PDB database [22] (Release from 01/07/2003) were clustered after removing redundant entries. These sequences were annotated using the SCOP database v. 1.63 [3,8]. The TRIBE-MCL results were calculated using the inflation value of 5 [5]. The total number of proteins used for analysis was 15,605 and 12,961 of the sequences had assigned SCOP domains. The total number of manually curated domains was 13,070 domains. Both annotated and non-annotated proteins were used for clustering. Obviously the method performance was calculated only for the annotated cases.

The SPC covered 6% fewer sequences for *K *= 20 but resulted in higher *a posteriori *specificity and sensitivities (Table 1). The TRIBE-MCL, however, resulted in higher *a priori *sensitivity. For this value of *K*, both the SPC and TRIBE-MCL clustered data into approximately the same number of clusters. Larger values of the parameter *K *further increased the number of covered sequences but decreased the performance of SPC. For example, an increase of *K *from 20 to the use of all connections ("all NN") covered an additional 330 sequences. The number of true positive predictions increased by 183. However, this increase was accompanied by an additional 173 false negative and 147 false positive predictions, thus decreasing the overall performance. Not all sequences were identical for both cases. The "20" clusters contained 300 sequences that were absent in "all NN" clusters. Correspondingly, there were 630 sequences that were present in "all NN" clusters but were absent in "20" clusters. The performance of the algorithm for these 630 new sequences was 569 true positives, 63 false negatives and 61 false positives. This corresponds to 90% in sensitivity and specificity.

Thus, the performance of the clustering method using all connections did not dramatically decrease due to the addition of the new sequences, but rather due to worse prediction of some sequences that were already clustered using *K *= 20. Therefore by joining results calculated using variable *K *values and preserving results calculated for the sequences that were clustered in each preceding step, one can expect to increase both sensitivity and specificity of the method. Indeed, the use of the gSPC method provided a considerable increase in clustering performance. The number of false positive and false negative for the "all NN" clusters was lower than the numbers calculated using fixed value of *K *= 20, but as many as 610 new sequences were covered. The gSPC method outperformed the TRIBE-MCL and SPC in terms of both *a priori *and *a posteriori *sensitivities. Thus the performance of the gSPC was considerably better than the other methods both in terms of covered sequences and quality of annotation. This improvement is of great importance for an automatic annotation of protein sequences.

### SwissProt analysis

The InterPro domains (v. 6.1) covered 112,935 (89%) sequences from the SwissProt database Release 41.9 (126,798 sequences). The total number of 235,672 domains was calculated for this set. The TRIBE-MCL clustered 97,792 sequences into 6,200 clusters that contained at least 4 annotated proteins (Table 2). The consensus annotation provided 93.8% of specificity for these data. A similar specificity of SPC, 94.1%, was calculated for *K *= 20 nearest neighbors. The number of covered sequences by the SPC algorithm was 96,716. The use of the gSPC made it possible to cover about 7,000 additional sequences with overall specificity and " *a posteriori*" sensitivity of 95.2% and 94%, respectively.

The SwissProt database provides a controlled vocabulary of 878 keywords that has been used by many researches to test different classification algorithms. The total number of 490,065 keyword instances was assigned to 125,248 proteins. As for the analysis of the InterPro domains, the gSPC analysis provided higher performance and covered a larger number of protein sequences compared to the use of any single *K*-value or TRIBE-MCL algorithm (Table 3).

### Comparison of algorithms for annotation of bacterial genomes

For this analysis 11,502 protein sequences from four completely sequenced genomes, *Bacillus subtilis*, *Helicobacter pylori*, *Listeria innocua *and *Listeria monocytogenes*, were used. The annotation of the genomes was done at MIPS using FunCat 1.3 [17]. The FunCat is an annotation scheme for the functional description of proteins from prokaryotes, unicellular eukaryotes, plants and animals [17,18]. Taking into account the broad and highly diverse spectrum of known protein functions, FunCat consists of 28 main functional categories (or branches) that cover general fields like cellular transport, metabolism and signal transduction. The main branches exhibit a hierarchical, tree like structure with up to six levels of increasing specificity. In total, the FunCat 1.3 included 1,445 functional categories and a total number of 403 distinct categories were available for analyzed bacterial genomes. The manual functional classifications were presented for 6,354 proteins.

An estimation of performance for proteins that have similar but not exact annotation represent some challenge. Let us consider an example of a cluster containing three proteins. The first protein that has annotation

01.01.01.01.02, biosynthesis of the glutamate group

01.05.01, C-compound and carbohydrate utilization

40.03, cytoplasm

the second protein has annotation

01.01.01.01.02.01, biosynthesis of proline

40.03, cytoplasm

and the third protein is annotated with one category only:

01.01.01.01.02, biosynthesis of the glutamate group

The annotation of all three proteins is similar but the annotation of the second protein is more detailed for the metabolism (01) category while the first protein contains additional category 01.05.01. The third protein does not have an annotation category 40, subcellular localization.

We measured the performance of annotation by counting the number of all non-redundant subcategories, i.e. 01, 01.01, 01.01.01, etc. In the above example the consensus annotation of this cluster is 01.01.01.01.02 and 40.03 categories (including all their sub-categories). The number of *TP *annotations is 19 = 7 + 7 + 5. The number of *FN *is 3. These are sub-categories 01.05, 01.05.01, and 01.01.01.01.02.01, for the first and second proteins, respectively. There are also two *FP *annotations, 40 and 40.03 observed for the third protein.

The performance of the three algorithms is shown in Table 4. The total number of non-redundant subcategories for this analysis was 44,531. The methods calculated similar performance, but the gSPC algorithm covered a larger number of sequences. Therefore the performance of gSPC was remarkably higher in terms of *a priori *sensitivity.

Table 5 summarizes the comparison of the three methods in terms of covered sequences and total error. Overall, the use of the gSPC algorithm resulted in higher performance for all examples and covered a larger number of sequences.

## Discussion

We have described and demonstrated the use and performance of SPC and of its extension, gSPC, for the clustering of protein sequences using sequence similarity only. For the first time, the SPC algorithms for clustering of protein sequences was employed in a large-scale study. Our results confirm that this method is a valuable, reliable tool for the automatic functional classification of protein sequences.

The use of the step-wise clustering approach, gSPC, improved sensitivity and specificity of the original method and allowed us to get a higher accuracy compared to the TRIBE-MCL and original SPC algorithms using data sets from PDB and SwissProt databases. The performance of the gSPC for annotation of Swiss Keywords favorably compares with supervised approaches. For example, the percentage of sequences covered by gSPC for 93.7% (*K *= 6) and 93% (*K *= 20) specificity were 62% and 79% respectively. The first number is similar to that calculated by the supervised classification approach using the C4.5 algorithm, where 58% of sequences were covered with 94% of specificity [23]. Thus the SPC algorithm classification performance is comparable to the state-of-art supervised machine learning classification approach used by [23]. Notice, that the latter method used different sequence attributes, such as PFAM domain and PROSITE composition, and thus explicitly took into consideration the multi-domain organization of proteins. The SPC algorithm, contrary to that, used the sequence similarity only. This result indicates high prediction ability of the annotation using gSPC clustering.

The specificity of the gSPC algorithm using all connections ("all-NN"), 92.8%, is also slightly superior to the results of another supervised approach, the adaptive algorithm for automated annotation [24] that calculated 90.4%. These results, however, are indicative only since our and previous approaches are different with respect to their types (i.e. supervised and unsupervised ones). In addition similar but not identical datasets were used. For example, the performance of the adaptive algorithm [24] was tested using 500 probe sequences, randomly chosen from SwissProt while the performance of the C4.5 algorithm was tested using 10-fold cross-validation.

A significant advantage of unsupervised clustering approaches over the supervised ones is the ability of the former methods to detect as yet unobserved relations between proteins. Such results could be used to find new protein families that currently do not have functional annotations or have incomplete or inconsistent ones. The unsupervised methods are also not subject to the risk of overfitting problems. Overfitting impairs the predictive power of supervised approaches for samples that were not represented in the training set [25-27].

The gSPC resulted in higher sensitivity and specificity for all analyzed datasets. The use of *a priori *sensitivity made it possible to compare results of all methods using a fixed number of samples and made the comparison more straightforward. However, from a practical point of view, particularly for the implementation of annotation pipe lines of complete genomes the specificity and *a posteriori *sensitivity rather than *a priori *sensitivity are the most relevant factors of the automatic annotation. Indeed, it is preferable to annotate a smaller number of sequences in automatic mode with a higher accuracy than to annotate more of them with a lower accuracy. The sequences not automatically annotated by the algorithm can be annotated subsequently by complementary approaches or by careful manual analysis of domain structure of the sequences. Any attempt to increase the *a priori *sensitivity and thus cover a larger number of sequences by lowering specificity may result in an unacceptable performance for annotation purposes.

The SPC algorithm calculates hierarchical tree-structures of clusters for each *K *value. In our analysis we identified and considered only the leaf clusters. The upper part of the tree was ignored. Such analysis was possible since for the data analyzed in this study the largest number of protein sequences were clustered in leafs and only a few additional sequences could be still clustered considering the whole tree structure. For example, using *K *= 6 (Table 1 &2, SPC results) only 56 and 627 additional sequences could be clustered for the PDB and SwissProt data sets, respectively. These numbers corresponded to about 1% sequences in each database and only marginally influenced the method performance. The gSPC algorithm, as it was already mentioned in the Method section, clustered such sequences at higher *K *values. This improved its prediction performance compared to the original SPC method.

The performance of any algorithm analyzing sequence relations depends on the selection of the reference data sets. The composition of such references data sets could bias the performance, since in reality the selection of sequences from known genomes or databases is not a representative random sample from the sequence space. For example, SwissProt and SCOP databases are very often used as "a gold standard" for annotation and classification methods. However, these databases represent a biased selection and do not cover entire genomes. Therefore, analysis of the performance of clustering methods is biased by the composition of the reference data sets. For example, the gSPC decreased missassignments by 2–5% for these two sets. Since the error rate for clustered sequences from these data sets was about 10–15% (Table 5), the relative gain in the performance was 10–40%. In other words, the automatic annotation of sequences clustered with gSPC will make up to 40% fewer erroneous annotations (false positive or false negative annotations) compared to other methods. On the other hand, all three methods returned similar results for the bacterial genomes. For this set gSPC covered about 12% of additional sequences which is very important for genome annotation.

The gSPC method developed is fast. A complete analysis of PDB and SwissProt datasets using all *K *levels took on moderate PC computer (AMD 1.3 GHz) less than 14 and 120 minutes, respectively. The speed of the original SPC algorithm scales approximately linearly with the number of connections. This number increases approximately as *N*^2 ^with the number of samples. However, since gSPC uses step-wise clustering, the actual number of samples remaining for clustering using large *K *values is small. Therefore, the gSPC speed is mainly determined by clustering using small, *K *= 2–6, values and is in practice approximately linear with the number of samples. In fact, the method will be fast enough to efficiently cluster datasets with millions of sequences; clustering computational requirements is therefore small compared to the computation of the sequence similarity scores. The computational efficiency is clearly an advantage of the gSPC method compared to the TRIBE-MCL. The complexity of the later scales as O(*N*^2^) were *N *is number of non-zero elements in the matrix. Therefore, this method performs slower if an increasing volume of genomic data needs to be processed.

## Conclusion

The gSPC calculated with higher accuracy or covered a larger number of sequences than the TRIBE-MCL algorithm for the analyzed datasets. The accuracy of annotation of gSPC for the SwissProt database was comparable to that of supervised methods. Thus it is a useful approach for automatic detection of protein families and annotation of full genomes.

## Methods

### Clustering of sequences using the properties of super paramagnetic systems

SPC performs a hierarchical clustering strategy [13]. The input data for SPC are represented as a distance matrix *d*_*ij *_(specified by the user) between data points *i = 1,...,N*. This matrix is used to construct a graph, whose vertices are the data points and edges corresponding to the connections between neighboring points. Each two points are considered to be neighbors (and thus have an edge), if they are within *K *nearest neighbors of each other (*K *-mutual-neighbor criterion). In the ferromagnetic model each point is considered to have a Potts spin, equivalent to one of *q *integer values, *s *= *1,2,...q*. Pairs of neighboring points *i *and *j *that have the same spin *s*_*i *_= *s*_*j *_are interacting with strength *J*_*ij*_, which is a function of an initial distance matrix *d*_*ij *_[13].

A Monte Carlo simulation using the Swendsen-Wang method (SW) [28] is used to determine clusters of points. The simulation starts with assigning a random Potts spin to each data point in the dataset. The neighboring points with identical spins interact with each other. The probability that these points belong to the same SW cluster (i.e., instant cluster resulting from an iteration) is calculated as *P*_*ij *_= (1 - exp(-*J*_*ij*_/*T*)), where *T *is some fixed temperature. The points from the same SW cluster receive identical spin (selected by chance) for the next iteration. The iterations are performed until convergence observed using, e.g. autocorrelation time [29].

For clustering, the strengths of the edges of the network are calculated using the spin-spin correlation function *G*_*ij*_. This function is estimated as the ratio of iterations when points *i,j *belong to the same SW cluster versus the total number of iterations during the simulation. Notice, that if *P*_*ij *_values determine an instant probability of two points with the same spin to belong to the same instant cluster (i.e., local connectivity), the spin-spin correlation function takes into account the multiple interactions amid points, i.e. global connectivity of the network. Actually the global connectivity determines the probability two points will have the same spin. At very low temperatures even small *J*_*ij *_cause neighboring points to have the same spin, *G*_*ij *_≅ 1, i.e. the system is in the ferromagnetic state. If the temperature is very high then all *J*_*ij *_<<*T *and the probability of finding each pair of neighboring points in the same state decreases to the value expected by chance *G*_*ij *_≅ *1/q*. Thus the system is unordered, i.e. it is in the paramagnetic state. If coupling values are equal *J*_*ij *_= *J*, only two these states are stable. The system has a sharp transition from the ferromagnetic to the paramagnetic state at a certain temperature.

In case of an inhomogeneous system, some magnetic "grains" could be observed. Such clusters of points have strong connections between the members and only weak connections to all points outside the cluster. Each grain has its own transition temperature from the ferromagnetic to the paramagnetic state. At some temperature range, the so-called superparamagnetic state, the system can contain some points in a locally ordered ferromagnetic state and as well as others in a locally unordered paramagnetic state. At the end of simulation, all points that had spin-spin correlation *G*_*ij *_> 0.5 form one cluster. The points that had *G*_*ij *_< 0.5 can be connected to their neighbors with maximal *G*_*ij*_. The results of the analysis at different temperatures are combined and provide a hierarchical clustering of data. A more detailed description of the SPC can be found elsewhere [13].

### Selection of free parameters of SPC algorithm

Free parameters of the SPC algorithm include the number of spins *q *in the Potts model, the number of nearest neighbors *K *for the *K*-mutual-neighbor criterion, and the number of iterations *I *for the SW algorithm. An increase of *q *required larger numbers of iterations for convergence but otherwise did not affect the performance of the algorithm [30]. For *q *= 20 the convergence of SW algorithm was usually observed using *I *= 1000 iterations for the vast majority of the cases investigated. The same values also resulted in convergence of the algorithm for the datasets used in our study.

As described above, SW simulations start with random spin configuration. After subsequent iterations, the algorithm converges into a stable state that does not depend on the initial configuration. Given convergence at a certain temperature, one can expect that the configuration should be similar to the converged state of the next step in respect to the increased temperature. Thus if SW simulations are started from a converged state calculated for a similar temperature, the convergence to the new state should be faster. Without loss of precision, we used this fact to speed up SW simulations. The configuration after completion of each simulation cycle was saved and used it as the initial one for the next temperature. It was possible to decrease the number of iterations from *I *= 1000 to *I *= 200 without affecting the quality of the results.

### Global clustering and the gSPC algorithm

The *K*-mutual-neighbor criterion controls the resolution of the clustering. A low *K *value takes into consideration only the closest pairs of mutually connected points ("i.e. short connections"). As its values increases, long-range connections are also taken into consideration and they participate in the clustering process. In previous studies this parameter was selected in the range of 5–20 [13,30]. In a recent study [31] an algorithm to determine optimal *K *values was also proposed. The optimal value of *K *corresponds to the maximum stable partitions characterized by maximum widths of the superparamagnetic domains. The optimization of *K *is critical in absence of reliable classification information to allow for supervised learning. In this study, reliable protein family assignment for test and training data was available. Thus, it was possible to directly analyze the performance of the SPC algorithm as a function of the parameter *K*.

Using a constant value for *K *is reasonable if the data set is rather homogeneous. In this case, the data points have the same connectivity on average. In reality, the problem of sequence based family assignment is complex. Some protein families apparently underlay constraints concerning the variation of their sequence and contain a large number of highly similar sequences while other families contain only few members displaying low sequence similarity. Using *K *values optimal for conserved families may bias the clustering process and lower the sensitivity to discover the more diverging ones. Thus, it is inappropriate to use a single *K *-value for clustering of all data.

In this article we introduce a step-wise analysis using different *K *-values. The data were consequently clustered using *K *= 2, 6, 20, 64 and *K *= "all" connections. This choice of *K *approximately corresponded to a 10-fold increase in the data connectivity that is proportional to *K*^2^. For each *K *we varied temperature from a minimum, *T *= 10^-4^, to a maximum, *T *= 2, values. At the maximum value of *T *no cluster was observed. As soon as a subset of sequences was clustered using some *K*, they were removed from any further analysis except one representative sequence. The representative sequence was the one that had the minimal sum of distances to the other sequences in the cluster. These representative sequences as well as all other remaining non-clustered sequences were used for the next round, i.e. larger *K*, of analysis. Such bottom-up clustering allowed us to cover different types of clusters covering a wide range of homogeneity and to improve the performance of the method as shown in the Result section. In order to distinguish the initial SPC algorithm from the step-wise based approach proposed in this study, we will refer to the latter as the global SPC (gSPC).

### Processing of hierarchic data

The SPC algorithm provided a hierarchical clustering of data on each step of the gSPC analysis. Let us consider the clustering procedure of increasing the temperature. For *T *= 0 all sequences form one cluster that starts to melt and break into subclusters as the temperature increases. Clusters of different sizes can appear and then melt at higher temperatures. Some clusters may contain only 2–3 sequences while other clusters consist of more than a thousand sequences. A number of clusters will lose one or several members for some particular temperatures while others will break into two or more sub-clusters. In case the data set contains only few clusters, one can inspect the cluster size or susceptibility, that is the variance of magnetization of the system as a function of temperature [13,30]. If different stable sub-phases are determined, e.g. by changes in the cluster sizes, it is possible to select a particular temperature in each region of interest for further analysis. Such a method, however, is unfeasible for the purpose of our study when as many as thousands clusters can be simultaneously observed for each particular temperature. Thus, the cluster composition for each temperature step should be considered for the analysis and some automated approach for the analysis of such results should be performed. In the following paragraphs we describe a method that performs such an analysis in a completely automatic way.

Let us limit a minimal size of any cluster to some parameter *C*. All points in clusters that have size less than *C *members were considered as unclustered points or singletons. The singletons did not form separate clusters but belonged to the cluster from which they were generated. At some particular temperature a cluster can break (melt) into a number of subclusters and singletons. However, if and only if these subclusters contained at least two subclusters each having at least *C *sequences, the parent cluster was considered to be subdivided into subclusters. Otherwise the cluster was considered to be unchanged. A leaf cluster was a cluster that did not form subclusters (i.e., it broke into singletons). The singletons were clustered again by the same procedure for larger *K*.

**Figure 2:**
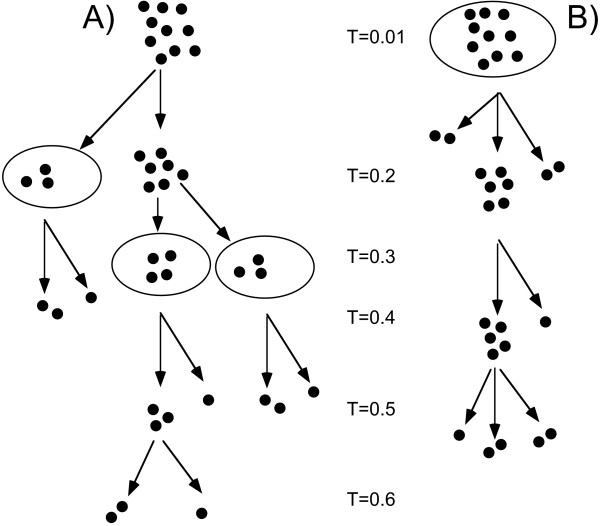
Detection of leaf clusters shown as ellipsoids. The minimal number of points in the leaf cluster is *C *= 3. The initial clusters contain 10 points each at *T *= 0.01 for both panel A) and B). On panel A) the cluster melts on two leaf clusters shown by ellipses. On panel B) the leaf cluster is the initial cluster. The leaf clusters are identified at different temperatures.

**Figure 3:**
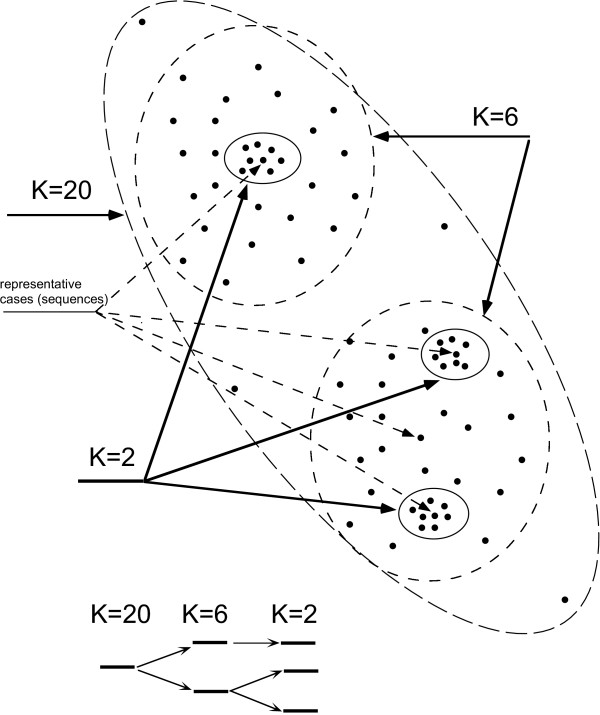
gSPC processing of simulated data using different *K*. The clusters detected for different *K *are shown as ellipsoids. On the first level *K *= 2, three most compact clusters are detected by the algorithm. The representative cases from these clusters will stay for further step of analysis, *K *= 6. For *K *= 6 two clusters are detected and, eventually for *K *= 20 one cluster will be detected. Thus the gSPC algorithm detects embedded clusters. The representative cases are used to restore hierarchical organization of clusters shown at the bottom of the figure. Notice, that for the upper clusters, the same sequence is the representative one for *K *= 2 and *K *= 6.

In other words, the result of the SPC clustering using a single *K *was a hierarchical tree of clusters (or several disjoined trees) generated by analyzing the data points over the whole temperature range (from minimal to maximal temperature). The tree was processed to detect the leaf clusters, which were identified at different temperatures. Figure 2 illustrates an example of the detection of leaf clusters for a single *K *and Figure 3 demonstrates processing of data for different *K*.

**Figure 4:**
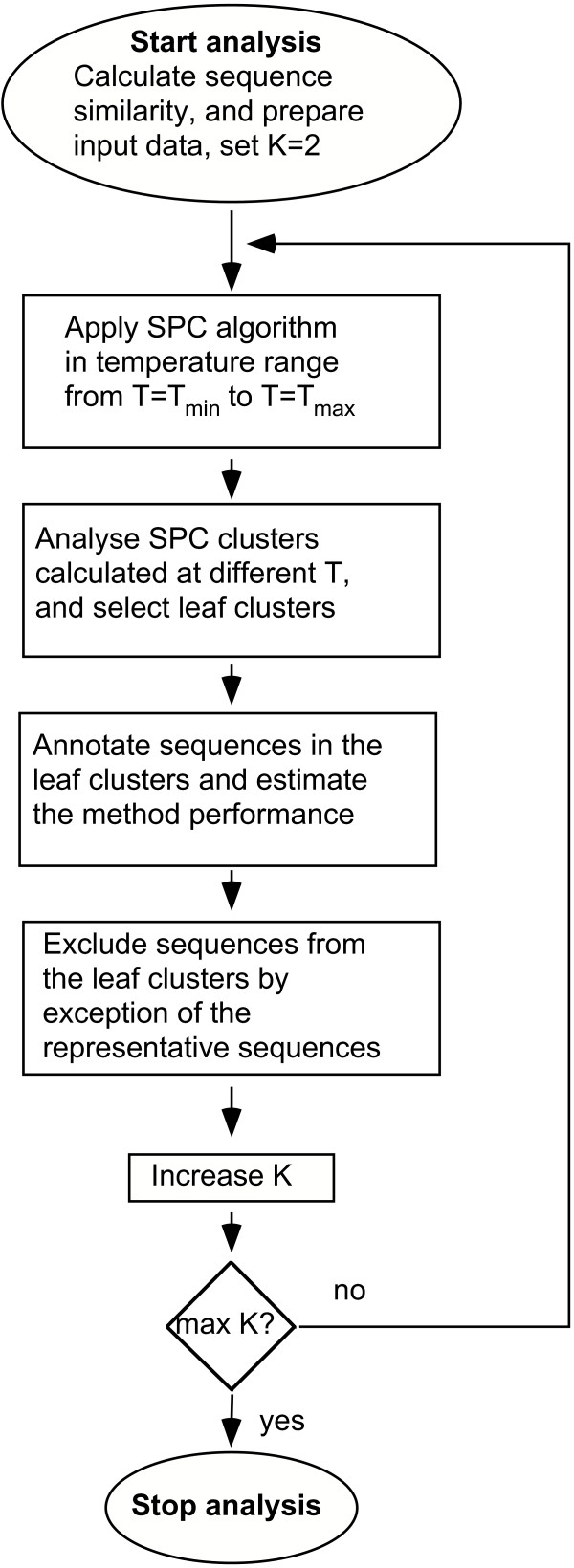
Data-flow of the gSPC algorithm.

The data flow of the algorithm (see Figure 4) is summarized as follows: In the very beginning the sequence similarities are downloaded from the SIMAP database as FASTA scores. Then, the algorithm clusters sequences using *K *= 2. During the analysis the temperature is scanned from a minimum *T *= 10^-4 ^(when practically all points form one large cluster) to a maximum *T *= 2 value (when almost all points are singletons). The leaf clusters are detected and used to calculate the performance of the algorithm for this *K *(see Tables 1,2,3,4). The sequences from the leaf clusters are then eliminated from further analysis except the representative ones (see above). Subsequently, we increase *K *and perform the next round of analysis. The analysis is terminated when the maximal specified *K *value is reached. The representative sequences may be assigned to some clusters with different *K *in later rounds of iteration. The annotation of these sequences in the cluster with smallest *K *was used to count the method performance. The gSPC procedure, as it was correctly noticed by the anonymous reviewer, also depends on the temperature step used to analyze clusters for each single *K*. For example, using large steps, ΔT = 0.3, one would detect different leaf clusters in Figure 2. As it is clear from the same figure, the sensitivity to this parameter is not a particular feature of gSPC but of the original SPC method that is used to determine the leaf clusters. Thus, the identification of leaf clusters can be ambiguous in some cases. In our article we used ΔT = 0.01 and did not observe significant changes in the performance of the algorithm for smaller values of this parameter.

### TRIBE-MCL algorithm

The TRIBE-MCL algorithm was downloaded from [32]. The values of the inflation parameter used for analysis of data were selected as indicated in the original study [5].

### Data representation

A FASTA file containing sequences that were used for clustering was compared to itself using BLAST [33] for the SwissProt database implemented in PEDANT [34]. For the bacterial genomes the FASTA pair-wise scores for were retrieved from the SIMAP database [10].

The all-against-all sequence similarities generated were parsed and used as input for both algorithms. The input data for TRIBE-MCL were represented as -log_10 _(*E-value*). The input values for the SPC were distance values calculated as -1./log_10 _(*E-value*). Pairwise scores with *E-value *> 0.1 were excluded from the analysis.

## List of abbreviations

PEDANT – Protein Extraction, Description and ANalysis Tool

FunCat – MIPS Functional Catalog

TRIBE-MCL – Markov Cluster Algorithm

SPC – Super Paramagnetic Clustering

gSPC – global SPC

SIMAP – Similarity Matrix of Proteins

## Authors' contributions

IVT programmed the algorithm and performed data analysis. AF developed graphical interface of the algorithm. AR annotated and provided the data for bacterial genomes and participated in their analysis. HWM conceived and supervised the project. All authors read and approved the final manuscript.

## Figures and Tables

**Table 1:** Clustering of PDB sequences using SPC, gSPC and TRIBE-MCL algorithms

*K*	Cases	clusters	true positive	false positive	false negative	specificity	*a posteriori *sensitivity	*a priori *sensitivity
SPC								
2	2472	479	2466	46	18	98.2	99.3	18.9
6	7332	1079	7107	276	274	96.3	96.3	54.4
20	8666	875	8324	413	401	95.3	95.4	63.7
all NN^1^	8996	740	8507	586	548	93.6	93.9	65.1
TRIBE-MCL								
	9208	964	8654	510	614	94.4	93.4	66.2
gSPC								
6	7432	880	7252	277	239	96.3	96.8	55.5
20	8961	233	8709	377	314	95.9	96.5	66.6
all NN^1^	9276	28	9009	392	329	95.8	96.5	68.9

1- the SPC analysis was performed using the complete similarity matrix and thus all Nearest Neighbors (NN) participated to the algorithm training.

**Table 2:** Analysis of InterPro domain composition in clusters calculated for SwissProt database

*K*	cases	clusters	true positive	false positive	false negative	specificity	*a posteriori *sensitivity	*a priori *sensitivity
SPC								
2	18960	4423	39414	1239	988	96.7	97.6	16.7
6	78441	11418	159201	6045	8474	96.3	94.9	67.6
20	96716	6635	185012	11514	20464	94.1	90.0	78.5
64	98568	3739	170364	18864	36143	90.0	82.5	72.3
all NN	91452	3420	155239	16206	32472	90.6	82.7	65.9
TRIBE-MCL								
	97792	6755	191500	12764	16939	93.8	91.9	81.3
gSPC								
6	79406	9543	162210	6208	7709	96.3	95.5	68.8
20	100458	2803	201766	9634	12402	95.4	94.2	86.6
64	103585	427	207131	10312	13264	95.3	94.0	87.9
All NN	103729	30	207339	10362	13304	95.2	94.0	88

**Table 3:** Analysis of SwissProt keywords composition in clusters calculated for the SwissProt database

*K*	cases	clusters	true positive	false positive	false negative	specificity	*a posteriori *sensitivity	*a priori *sensitivity
SPC								
2	19161	4473	78782	4421	3373	94.7	95.9	16.1
6	79642	11643	308068	20598	261446	93.7	92.2	62.9
20	98276	6875	354980	35282	53628	91.0	86.9	72.4
64	100177	3953	331422	47215	80772	87.5	80.4	67.6
all NN	93601	3601	308433	41834	74472	88.1	80.6	62.9
TRIBE-MCL								
	99636	7015	364554	36783	49333	90.8	88.1	74.4
gSPC								
6	80617	9755	314537	21080	23838	93.7	93.0	64.1
20	101805	2857	388434	29379	35042	93.0	91.7	79.3
64	105248	445	400100	31061	37325	92.8	91.5	81.6
all NN	105339	19	400363	31142	37391	92.8	91.5	81.7

**Table 4:** Clustering of sequences of bacterial genomes using SPC, gSPC and TRIBE-MCL algorithms

*K*	cases	clusters	true positive	false positive	false negative	specificity	*a posteriori *sensitivity	*a priori *sensitivity
SPC								
2	646	157	5365	357	99	93.8	98.2	12.1
6	4652	794	34699	2635	2624	92.9	93.0	78.2
20	5072	637	35862	3840	5396	86.9	90.3	80.1
all NN	4993	592	34375	4160	6241	89.2	84.6	77.5
TRIBE-MCL								
	4517	704	34563	2475	3042	93.3	91.9	77.9
gSPC								
6	4612	710	34631	2472	2365	93.3	93.6	78.2
20	4988	115	37574	2836	2948	93.0	92.7	84.7
All NN	5043	18	37862	2968	3105	92.7	92.4	85.4

**Table 5:** Comparison of different clustering algorithms

Analyzed data set	SPC		MCL		GSPC	
	
	clustered cases	error,^1 ^%	clustered cases	error,^1 ^%	clustered cases	error,^1 ^%
SCOP domains	8666 (94%)^2^	9.3	9208	12.2	9276 (101%)	7.7
SwissProt InterPro domains	96716 (99%)	15.6	97792	14.3	103729 (106%)	10.7
SwissProt keywords	98276 (99%)	21.8	99636	20.8	105339 (106%)	15.7
Bacterial genomes, FunCat 1.3	4652 (103%)	14.1	4517	14.7	5043 (112%)	14.8

1-The error is defined as error=100%*(FP+FN)/(TP+FN). 2-In parentheses the percentage of clustered sequences relative to the corresponding numbers of the MCL algorithm (100%) are indicated.
